# Effect of Hardening Exponent of Power-Law Hardening Elastic-Plastic Substrate on Contact Behaviors in Coated Asperity Contact

**DOI:** 10.3390/ma11101965

**Published:** 2018-10-12

**Authors:** Xiqun Lu, Hanzhang Xu, Bin Zhao

**Affiliations:** College of Power and Energy Engineering, Harbin Engineering University, Harbin 150001, China; luxiqun@hrbeu.edu.cn (X.L.); xuhanzhang@hrbeu.edu.cn (H.X.)

**Keywords:** asperity contact, coatings, power-law hardening, contact behaviors, contact mechanics

## Abstract

The contact between a rigid flat and a coated asperity is studied using the finite element method. The substrate is assumed as the power-law hardening elastic–plastic material. The effect of the hardening exponent of the substrate (*n*) on the contact behaviors including contact load, area, coating thickness variation and stress in the coating, is investigated. It shows larger hardening exponent results in larger contact loads and larger maximum stresses in the coating at a given interference, and leads to smaller contact area at a specific contact load. The coating thickness becomes smaller monotonically as the interference increases for larger hardening exponents, while it recovers gradually after reaching the minimum value for the smaller *n* cases. This work will give some universal guidance to improve the contact performance for coatings by adjusting the hardening exponent of the substrate and by optimizing the coatings parameters.

## 1. Introduction

Contact between surfaces plays a very important role in the industry field [1]. Coatings have been widely applied on the contact surfaces [2,3,4], since it is an effective means to improve the mechanical performance such as the wear resistance, corrosion resistance and so on, where tribological performance should be specially considered among them. To select optimal coatings efficiently and achieve best tribology performance for different substrate materials is very meaningful but full of challenges. Until now, the trial and error method is still the usual approach since there seems to be little effective and perfect theories to guide to achieve the coating optimization, as the authors know. Therefore, to develop the related theory should be the direction which guides the efforts going forward.

All contact surfaces are rough on the microscale and contain many asperities. When two coating surfaces are in contact with each other, the contact happens at the top of the coated asperity in fact. Therefore, the contact between coated asperities should be the first problem to be considered, which is always simplified as the contact between a sphere and a flat [5]. Generally, when an asperity and a flat contact with each other, the asperity could be flattened by the flat or indent it [6]. Most studies about the coating contact so far considered the indentation, i.e., a rigid spherical indenter contact with a coated deformable flat, since they focused on the mechanical properties of the coating. Pogrebnjak [7] studied the structural and mechanical properties of NbN and Nb–Si–N films with the experimental method and the molecular dynamics simulation. Coy et al. [8] considered the topographic reconstruction and mechanical properties of atomic layer deposited Al_2_O_3_/TiO_2_ nanolaminates by nanoindentation. However, when the tribological performance is the focus of attention, the flattening that is associated with mild adhesive friction and wear should be considered. In this work, the flattening process of the coated asperity by a rigid flat is explored.

Chang [9] developed a modified elastic–plastic asperity contact model on basis of Greenwood–Williamson (GW) model [10] to study the contact between surfaces with soft coatings. The effect of the coating thickness and plasticity index on the contact area and normal load was investigated. McCool [11] described a micro-contact model by extending the GW model [10] to investigate the coating contact, and explored the effect of the coating thickness and compliance on the contact load, area and mean real pressure. Liu et al. [12] presented extended Hertzian formulae to predict contact behaviors for circular and elliptical point contact problems with the presence of coating. They expressed the extended equivalent modulus of the coated asperity as the function of the coating thickness along with Young’s modulus and Poisson’s ratio of the coating and the substrate. Garjonis et al. [13] presented the analytical and finite element (FE) models by means of Hertz theory and FE methods respectively, to study the normal contact between two layered spheres in the elastic phase. They obtained the force-displacement relation in the process of contact between the layered asperities, and validated these relations by FE results. Yeo et al. [14] used an improved elastic contact model to investigate the contact between a coated asperity and a rigid flat, and to study the asperity contact stiffness. The results were also verified by the FE results. Goltsberg and Etsion [15] used the FE method to explore the elastic contact between a coated asperity and a rigid flat. They studied the effect of the geometrical and mechanical properties of the coating and substrate on contact behaviors, and got universal dimensionless formulae about the relation of load and displacement based on many simulation results. Subsequently, Goltsberg and Etsion [16] continued to study the contact area and maximum equivalent stress using the FE method and obtained the corresponding dimensionless expressions, constituting a complete universal contact model for the elastic coated asperity contact along with their previous work presented in [15].

All these models about the coated asperity contact introduced above considered the elastic case, i.e., both the substrate and the coating were assumed as the purely elastic materials. However, for many metallic materials, their stress-strain relations actually show elastic–plastic characteristics. Therefore, it should be necessary to consider the behavior of the elastic–plastic asperity contact. Eid et al. [17] proposed a coated asperity contact model considering the effect of adhesion. The material properties of the asperity including the substrate and coating are both assumed to be elastic–plastic with 2% linear hardening of the elastic modulus. The effect of the coating thickness on the pull-off force and contact radius in the loading and unloading processes was studied. Goltsberg et al. [18] investigated the onset of the plastic yielding in a coated sphere when it contacted with a rigid flat by means of the FE software. The asperity materials were also assumed as elastic–plastic with 2% linear hardening of the elastic modulus. The influence of the asperity material properties and the coating thickness on the critical contact parameters was investigated, such as critical normal load, area and interference when the plastic yielding first appears. Chen et al. [19] continued to study the plasticity evolution in the coated asperity which is 2% linear hardening elastic–plastic material during the process of contact with a rigid flat, and obtained empirical expressions about the critical interferences at the onset of plastic yielding. In addition, they found that as the interference increased, the elastic core (low-stress zone) appeared under the contact area, leading to the decrease of the mean pressure.

Most of the FE models mentioned above about the coated asperity contact focused on the elastic or 2% linear hardening elastic–plastic asperity materials. However, for many materials, the power law is more precise to describe their strain hardening behavior [20]. The influence of the power-law hardening material properties on the coated asperity contact has not been studied in detail, which will be considered in this work where the substrate is set as the power law hardening elastic–plastic materials. Then, the effect of the substrate-hardening exponent on the contact parameters such as contact load, area and stress is focused on in this work. On basis of the numerical analysis in this work, the effect of the material property and geometrical characteristics of coating/substrate system on its tribological performance could be predicted preliminarily and accurately in the future, and then it would be meaningful to help choose the optimal coating and obtain the best tribological performance. It should be noted that this work focuses on the very hard coating such as diamond-like carbon (DLC) film, which could be simplified and assumed as the purely elastic materials as revealed in Refs. [15,16].

## 2. Construction of the Finite Element Model

### 2.1. Power-Law Hardening Property

**Figure 1:**
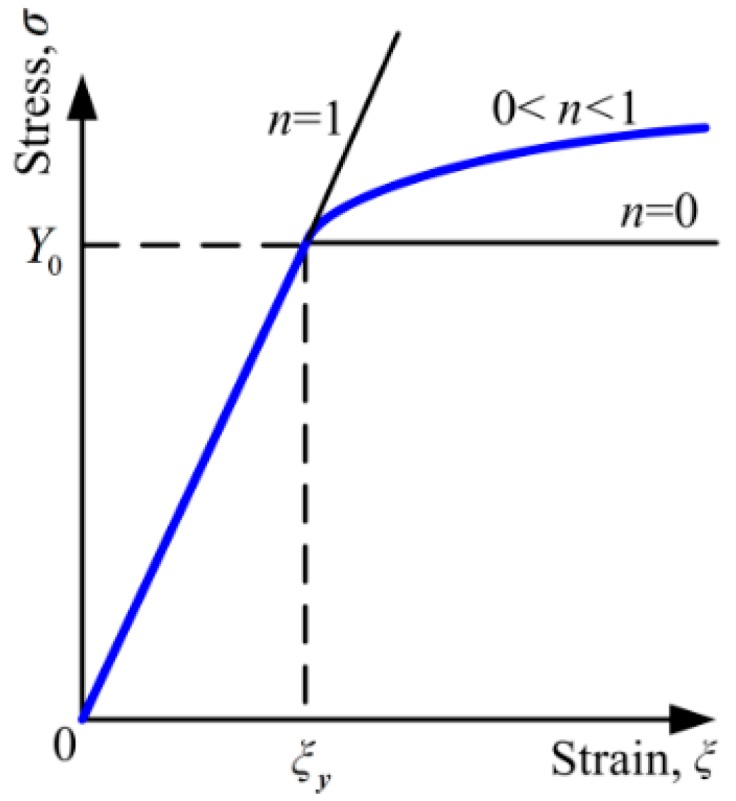
The constitutive law of power-law hardening elastic–plastic materials.

In this work, the substrate is assumed as the power law hardening elastic–plastic material. The relation between the stress and strain at first are linear in the elastic stage, while after the yield strength *Y*_0_, i.e., in the hardening stage, the relation changes to be power law and the plastic behavior satisfies *J*_2_ flow theory and isotropic hardening model, as shown in Figure 1. The relation is descripted as Equation (1).

(1) $$\xi =\left\{ \begin{array}{l} \sigma /E,\sigma \leq Y_{0}\\ (Y_{0}/E)(\sigma /Y_{0}){}^{1/n},\sigma >Y_{0} \end{array}\right.$$

where *ξ* is the strain, *σ* is the stress, *E* is the Young’s Modulus, *Y*_0_ is the yield strength, and *n* is the strain hardening exponent ranging from 0 to 1. For two extreme cases, *n* = 1 or *n* = 0, the materials change to be the purely elastic or elastic perfectly-plastic case.

### 2.2. Finite Element Model

**Figure 2:**
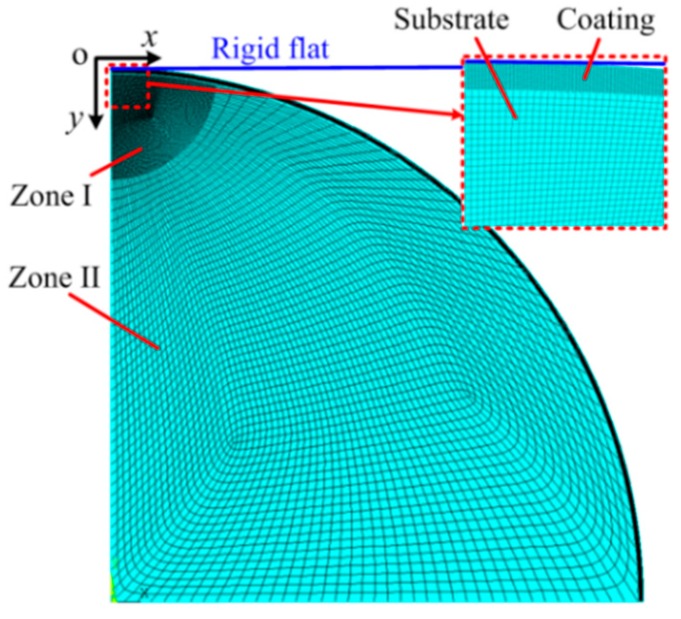
The two-dimensional finite element model.

A two-dimensional axisymmetric finite element model was developed to consider the contact between a rigid flat and a coated asperity using the software ANSYS 16.1, as presented in Figure 2. The asperity was modeled as a quarter of a circular plane, consisting of the coating part whose thickness was *t* and the substrate part whose radius was *R*. The rigid flat was represented by a line. Considering the contact region is of most interest, the coating part and the substrate part close to the contact region (Zone I in Figure 2) were meshed fine. The radius of Zone I is 0.1 *R*. Zone II of the substrate part was discretized much more coarsely since it was far away from the contact region. The eight-node PLANE 183 element was used to mesh the asperity, while the contact element CONTA 172 and target element TAEGET 169 were utilized to consider the contact between the asperity and rigid flat. After meshing, the total number of the elements are 17,810 and the nodes are 48,825.

Two boundary conditions were assumed as follows: First, nodes on the symmetry axis (*y* axis in Figure 2) were limited to move in the radial direction; secondly, nodes at the bottom of the asperity were constrained in all directions. The frictionless contact condition between the asperity and the flat was used, while for the contact between coating and substrate, the bonded condition was adopted. The coating part was set as the elastic homogenous material without residual stresses, while the substrate part was assumed as the elastic power-law hardening plastic material. Both parts were isotropic. A displacement load was applied to the coated asperity through the rigid flat, and the reaction force of the rigid flat as an output could be obtained as the contact force. The von Mises yielding criterion was adopted to consider the behavior of substrate changing from elastic to plastic. The augmented Lagrangian method was selected as the solution method.

The developed FE model was verified with two following approaches. First, assuming the coating and substrate materials were identical and elastic, the contact forces were calculated by the developed FE model in this work and compared with the analytical Hertz solution. The differences were shown to be less than 2.8%. Secondly, the mesh density was doubled iteratively until it had little effect on the results between successive iteration, in order to ensure the mesh convergence.

It should be noted that some ideal assumptions were used in this FE model making the complicated contact problem simplified as follows: (1) The contact between the asperity and the rigid is frictionless; (2) the bonded condition is adopted between the coating and the substrate; (3) the substrate part is assumed as the elastic power-law hardening plastic material. Typical results could be obtained to preliminarily explore the effect of the power-law hardening elastic–plastic materials of the substrate on the contact behaviors. As the work goes on, the ideal assumptions will be relaxed step by step in the future.

## 3. Results and Discussion

The results about the contact behaviors obtained with the developed FE model are shown in this section. The geometrical and material parameters for the coated asperity are set as follows: The asperity radius *R* changes from 5 mm to 20 mm; the dimensionless coating thickness *t*/*R* varies in the range of 0.001 ≤ *t*/*R* ≤ 0.02 with the interval of 0.001; the Poisson’s ratios of the coating *ν*_co_ and the substrate *ν*_su_ are both set as 0.32, a common value for metallic materials; the Young’s modulus of the coating *E*_co_ and the substrate *E*_su_ changes in a wide range of 1 ≤ *E*_co_/*E*_su_ ≤ 10; the yield strength of the substrate *Y*_0_sub_ is assumed as a constant 210 MPa; the hardening exponent of the substrate *n* ranges from 0 to 1. The geometrical and material parameters could be chosen arbitrarily from the range mentioned above, covering a large number of realistic materials to consider the effect of the substrate hardening exponent on the contact behaviors. Some typical cases are selected and shown as follows based on the condition that asperity radius *R* is 10 mm.

**Figure 3:**
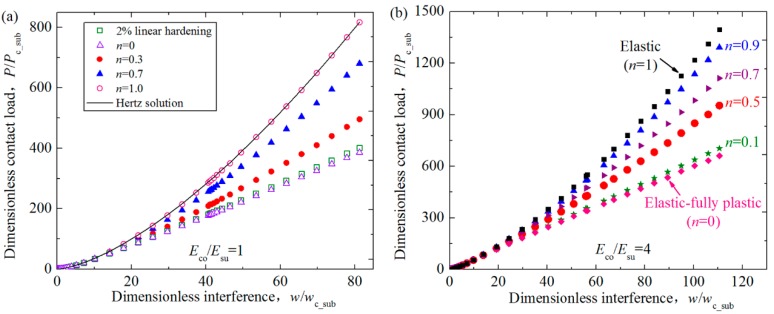
The relation between the dimensionless contact load *P*/*P*_c_sub_ and interference *w*/*w*_c_sub_ for some typical cases where dimensionless coating thickness *t*/*R* = 0.01, hardening exponents of the substrate *n* = 0.1, 0.5, 0.7, 0.9 and Young’s modulus ratios *E*_co_/*E*_su_ = 1, 4. The results for the purely elastic (*n* = 1) and elastic fully-plastic (*n* = 0) cases are also given and compared with existing literature. (**a**) *E*_co_/*E*_su_ = 1; (**b**) *E*_co_/*E*_su_ = 4.

Figure 3 presents the influence of hardening exponents of the substrate *n* on the relation between the dimensionless contact load *P*/*P*_c_sub_ and interference *w*/*w*_c_sub_ for the contact between a rigid flat and a coated asperity having the power law hardening elastic–plastic substrate. Some typical results are selected to be shown for the cases where the hardening exponent of the substrate *n* ranges from 0 to 1 at two selected Young’s modulus ratios *E*_co_/*E*_su_ = 1, 4. The dimensionless coating thickness *t*/*R* is set as 0.01. The critical contact area, load and interference of the substrate, *A*_c_sub_, *P*_c_sub_ and *w*_c_sub_, at the yielding inception under the normal load were given by Jackson and Green [21]:

(2) $$A_{c\_\mathrm{sub}}=\pi^{3}{\left(\frac{C_{\nu }Y_{0\_\mathrm{sub}}R}{2E_{\mathrm{su}}}\right)}^{2}$$

(3) $$P_{c\_\mathrm{sub}}=\frac{\pi^{3}Y_{0\_\mathrm{sub}}}{6}C_{\nu }^{3}{\left(R(1-\nu^{2})(\frac{Y_{0\_\mathrm{sub}}}{E_{\mathrm{su}}})\right)}^{2}$$

(4) $$w_{c\_\mathrm{sub}}={\left(C_{\nu }\frac{\pi (1-\nu^{2})}{2}(\frac{Y_{0\_\mathrm{sub}}}{E_{\mathrm{su}}})\right)}^{2}R$$

where *E*_su_ and *Y*_0_sub_ is the Young’s modulus and yield strength of the substrate, *C_ν_* is a parameter related to the Poisson’s ratio as $C_{\nu }=1.234+1.256\nu$.

Figure 3a shows the results of the contact load for *E*_co_/*E*_su_ = 1. Two extreme cases are also given as a comparison. One is the case of the substrate hardening exponent *n* = 1.0, which means the asperity transforms into the homogenous and purely elastic material. In this case, the contact load is calculated and compared with the Hertz solution. The differences are less than 2.8% as stated in Section 2. The other extreme case is that *n* = 0, which means the substrate changes to be the elastic perfectly-plastic material. The numerical results for 2% linear hardening substrate case are also shown in Figure 3a, to simulate the elastic perfectly-plastic material under the small interference considering its stress-strain relation as revealed in Ref. [22]. The results show a good coincidence besides a small difference. The difference gets larger as the interference increases. This is due to the reason that the difference of material properties between the real 2% linear hardening elastic–plastic material and the ideal elastic perfectly–plastic material gets larger. The comparison for these two extreme cases proves that the FE model is accurate and can be used to consider the contact behavior under other conditions. Figure 3b shows the results for *E*_co_/*E*_su_ = 4. The elastic (*n* = 1) and elastic fully-plastic (*n* = 0) substrate of the asperity are also considered, which appears at two sides of the different exponent cases (0 < *n* < 1). This also can prove the accuracy of this model to some extent. The curves in Figure 3 reveal that the load increases with the increase of interferences. In addition, for the same Young’s modulus ratio case, the load becomes larger as the hardening exponent increases from 0 to 1 at a given interference. This is because as the hardening exponent increases, the substrate becomes more elastic, which means the reaction force of the asperity is larger under the same interference.

**Figure 4:**
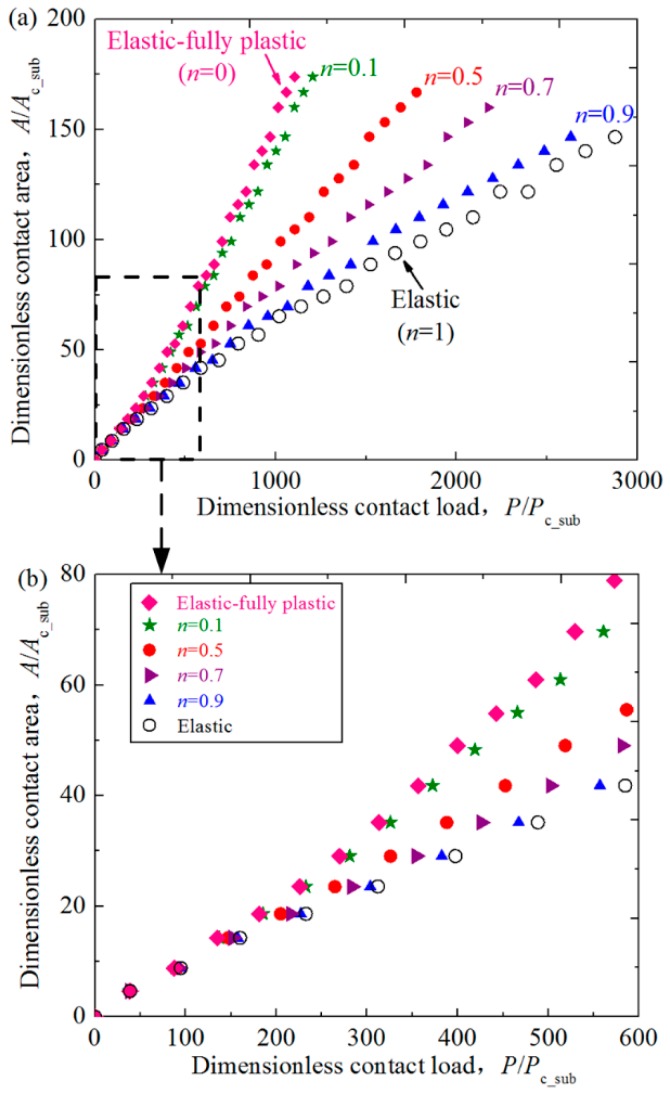
The relation between the dimensionless contact area *A*/*A*_c_sub_ and load *P*/*P*_c_sub_ for some typical cases where dimensionless coating thickness *t*/*R* = 0.01, Young’s modulus ratios *E*_co_/*E*_su_ = 4 at different hardening exponents of the substrate *n* = 0.1, 0.5, 0.7, 0.9. The results for the purely elastic (*n* = 1) and elastic fully-plastic (*n* = 0) cases are also given. (**a**) 0 ≤ *P/P*_c_sub_ ≤ 3000; (**b**) 0 ≤ *P/P*_c_sub_ ≤ 600.

**Figure 5:**
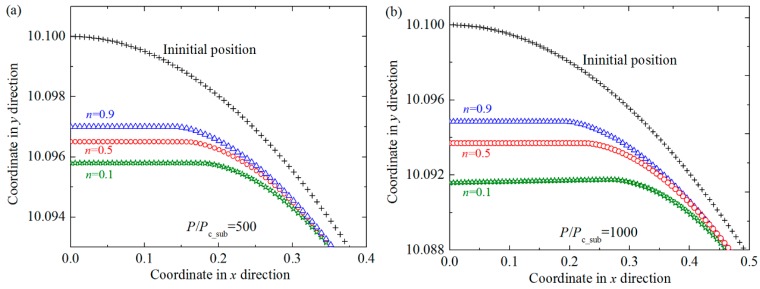
The profiles of the outer surface before and after the normal loading process for some typical cases where dimensionless coating thickness *t/R* = 0.01, Young’s modulus ratios *E*_co_*/E*_su_ = 4 at different hardening exponents of the substrate *n* = 0.1, 0.5, 0.9 under selected dimensionless contact loads *P/P*_c_sub_ = 500, 1000. (**a**) *P/P*_c_sub_ = 500; (**b**) *P/P*_c_sub_ = 1000.

The contact area plays a very important role in the tribological behaviors such as wear, friction and so on, and thus the effect of the hardening exponent of the substrate on the contact area of the coated asperity are also studied in this work. Figure 4 shows the relation between the dimensionless contact area *A*/*A*_c_sub_ and load *P*/*P*_c_sub_ for some typical cases where dimensionless coating thickness *t*/*R* = 0.01, Young’s modulus ratios *E*_co_/*E*_su_ = 4 at some selected hardening exponents of the substrate *n* = 0.1, 0.5, 0.7 and 0.9. In addition, the cases for the purely elastic (*n* = 1) and elastic fully-plastic (*n* = 0) substrate are also considered and shown in Figure 4. It could be seen that the contact area increases as the value of *n* decreases at a specific contact load. It is because the substrate with smaller *n* is more plastic, and the same contact load will lead to larger interferences. Thus the contact area increases accordingly. This tendency can also be seen from Figure 5, where the change of the profiles of the asperities with different substrate materials before and after the normal loading is given. The contact radii are shown as the flat parts in the center of the profiles, which also represents the size of the contact area. It could also be found that as the hardening exponent of the substrate *n* decreases, the contact radii become larger, which accords with the conclusions given in Figure 4.

**Figure 6:**
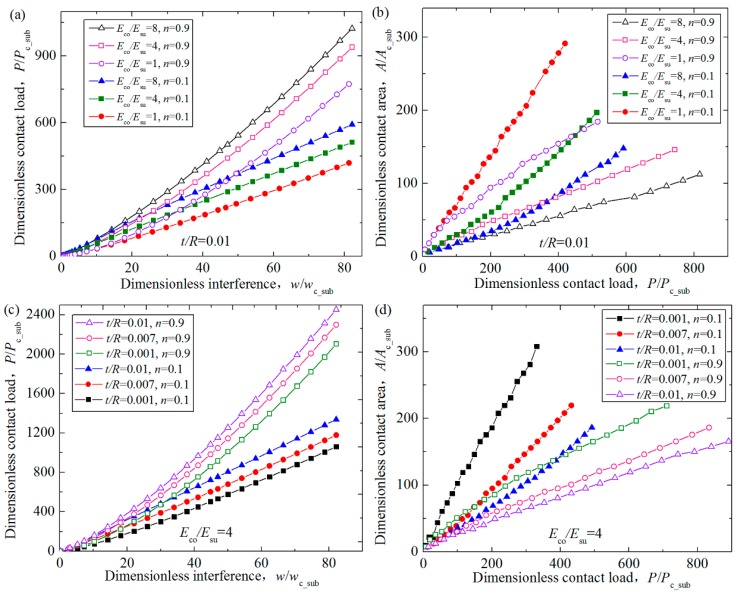
The effect of the Young’s modulus ratios and dimensionless coating thicknesses on the dimensionless contact load *P/P*_c_sub_ and area *A/A*_c_sub_ at different hardening exponents of the substrate *n* = 0.1, 0.9, where (**a**) *P/P*_c_sub_ vs. *w*/*w*_c_sub_ and (**b**) *A/A*_c_sub_ vs. *w*/*w*_c_sub_ are for the cases where the Young’s modulus ratios *E*_co_*/E*_su_ = 1, 4, 8 and the dimensionless coating thickness *t/R* = 0.01; (**c**) *P/P*_c_sub_ vs. *w*/*w*_c_sub_ and (**d**) *A/A*_c_sub_ vs. *w*/*w*_c_sub_ are for the cases where the dimensionless coating thickness *t/R* = 0.001, 0.007, 0.01 and the Young’s modulus ratios *E*_co_*/E*_su_ = 4.

The effect of the Young’s modulus ratios and coating thicknesses on the dimensionless contact behaviors was already considered in detail for the purely elastic or 2% linear hardening elastic–plastic asperity materials in the existing studies [15,16,19], and the corresponding variation trend has been gotten for these materials. While for the power-law hardening substrate considered in this work, the trend should be verified. Therefore, the effect of the Young’s modulus ratios and dimensionless coating thicknesses on the dimensionless contact load *P/P*_c_sub_ and area *A/A*_c_sub_ are investigated, and the results for some typical cases where the typical hardening exponents *n* = 0.1 and 0.9 are revealed in Figure 6. Figure 6a,b shows that the variation of contact parameters for the cases where the Young’s modulus ratios *E*_co_*/E*_su_ = 1, 4, 8 when the dimensionless coating thickness *t/R* = 0.01. It could be seen that for the coated asperity having a given *n* value, the contact load increases as the *E*_co_*/E*_su_ value increases at a given interference. In other words, the interferences were larger for the coating system with smaller *E*_co_/*E*_su_ values to get the same contact load. It means this kind of coating system has higher load bearing capacity to resist plowing, which will lead to smaller friction coefficient. This could help explain the experimental results shown in Figure 2 in Ref. [23], where the TiN(coating)/WC-Co(substrate) system presented a lower friction coefficient in the running-in stage since it had the lower elastic modulus ratio *E*_co_/*E*_su_ than the TiN(coating)/Cu(substrate) system [23]. While at a given contact load, the contact area decreases with the increase of the *E*_co_*/E*_su_ values. This trend is in accord with the conclusions given in Ref. [16]. Figure 6c,d show the effect of the dimensionless coating thickness *t/R* on the contact parameters. Some typical dimensionless coating thicknesses *t/R* = 0.001, 0.007, 0.01, spanning an order of magnitude, are considered for the Young’s modulus ratios *E*_co_*/E*_su_ = 4. As shown in Figure 6c,d, the contact load increases as the coating thickness becomes larger when the substrates are the same material. It is expected because the coating is harder and more elastic than the substrate, and the same interference will lead to larger contact load for the asperity with larger coating thickness. The contact area decreases with the increase of the coating thickness. It is probably because the same contact load will leads to less yielding for the asperities with the thicker coating. These results above accord with the conclusions given in Ref. [15].

**Figure 7:**
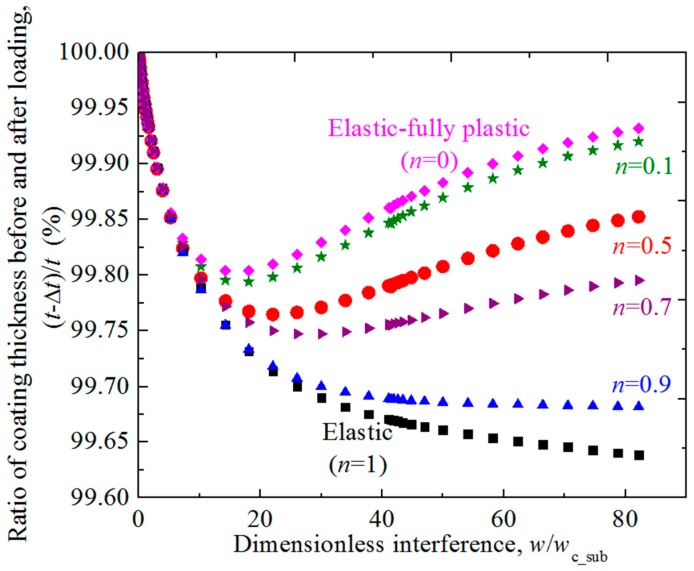
The ratio of coating thickness before and after loading (*t* − Δ*t*)/*t* changing with the dimensionless interference *w/w*_c_sub_ for typical cases where hardening exponent *n* = 0.1, 0.5, 0.7, 0.9 for dimensionless coating thickness *t/R* = 0.01 and Young’s modulus ratios *E*_co_*/E*_su_ = 4. The elastic (*n* = 1) and elastic fully-plastic (*n* = 0) cases are also considered.

The effect of the hardening exponent of substrate on the change of the coating thickness during the normal loading process is studied. Figure 7 shows the ratio of coating thickness before and after loading, (*t* − Δ*t*)/*t*, changes with the dimensionless interference *w/w*_c_sub_ for different hardening exponent *n* = 0.1, 0.5, 0.7, 0.9 cases. The decrease of the coating thickness on the axis of symmetry Δ*t* could be calculated as the difference value between the interference of rigid flat *w* and the axial displacement of the interface point on the axis of symmetry *w*_sub_, i.e., Δ*t* = *w − w*_sub_. The elastic (*n* = 1) and elastic fully-plastic (*n* = 0) cases are also considered. The dimensionless coating thickness *t/R* are set as 0.01 and Young’s modulus ratios *E*_co_*/E*_su_ = 4. It could be seen from Figure 7 that for the asperity with the elastic substrate (*n* = 1) or with the substrate whose hardening exponents are large and close to 1 (such as *n* = 0.9), the coating thickness becomes smaller monotonically as the interference increases in the loading process. For these cases, the rate of the variation of coating thickness (absolute value) is very large at the very beginning of the contact, and then decreases gradually as the interference increases until it approaches zero (see the case *n* = 0.9 in Figure 7). While for the asperity with the substrate whose hardening exponents are smaller (such as *n* ≤ 0.7), the coating thickness also reduces considerably in the early contact stage until reaches a minimum value, then it recovers gradually. For smaller hardening exponent cases, the transition point appears earlier and the recovery of the thickness is more obvious and rapid. Especially for the small *n* cases such as *n* = 0 and 0.1, the coating thickness can recover to 99.9% of the initial value.

**Figure 8:**
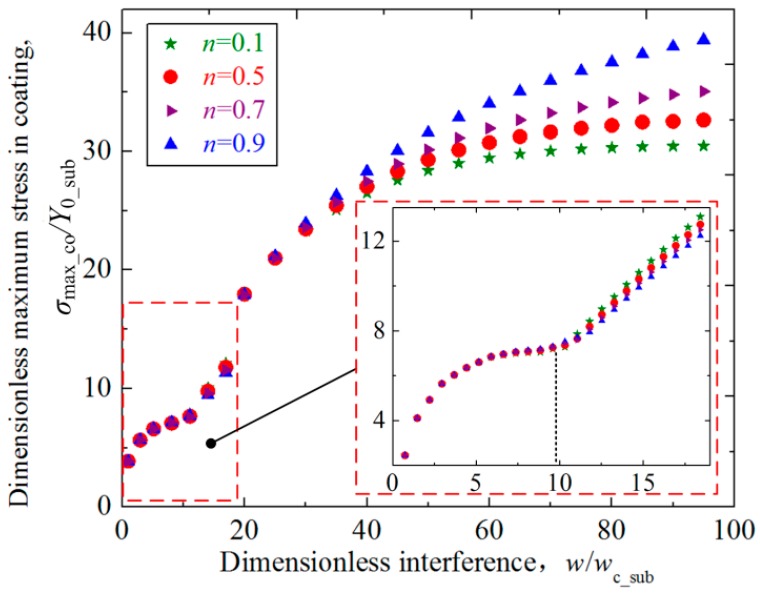
The relation between the dimensionless maximum von Mises stress *σ*_max_co_*/Y*_0_sub_ in the coating and the dimensionless interference *w*/*w*_c_sub_ for some typical cases where dimensionless coating thickness *t/R* = 0.01, Young’s modulus ratio *E*_co_*/E*_su_ = 4 at selected hardening exponents of the substrate *n* = 0.1, 0.5, 0.7, 0.9.

The distribution and intensity of the contact stress is closely related to the possible failure modes in the coated asperity contact, and the effect of the substrate hardening exponent on the stress should be investigated. Figure 8 shows the relation between the dimensionless maximum von Mises stress in the coating *σ*_max_co_/*Y*_0_sub_ and the dimensionless interference *w*/*w*_c_sub_ for some selected hardening exponents of the substrate *n* = 0.1, 0.5, 0.7, 0.9. The dimensionless coating thickness *t*/*R* = 0.01, and Young’s modulus ratios *E*_co_/*E*_su_ = 4. It could be seen from Figure 8 that the maximum stress increases as the interference *w*/*w*_c_sub_ becomes larger. Under the small interference, the effect of the substrate with different *n* values on the maximum stress in the coating is negligible. Then the dimensionless interference *w*/*w*_c_sub_ gets larger ranging from 12 to 40, the maximum stress still shows to be slightly influenced by the *n* values. While the interference *w*/*w*_c_sub_ is above 40, the effect of the *n* value on the maximum stress could not be omitted, and larger *n* values of the substrate result in larger maximum stresses in the coating. This is expected since the larger *n* values means more elastic substrate, and the same interference will lead to larger maximum stress for the asperities with these kind of substrates.

**Figure 9:**
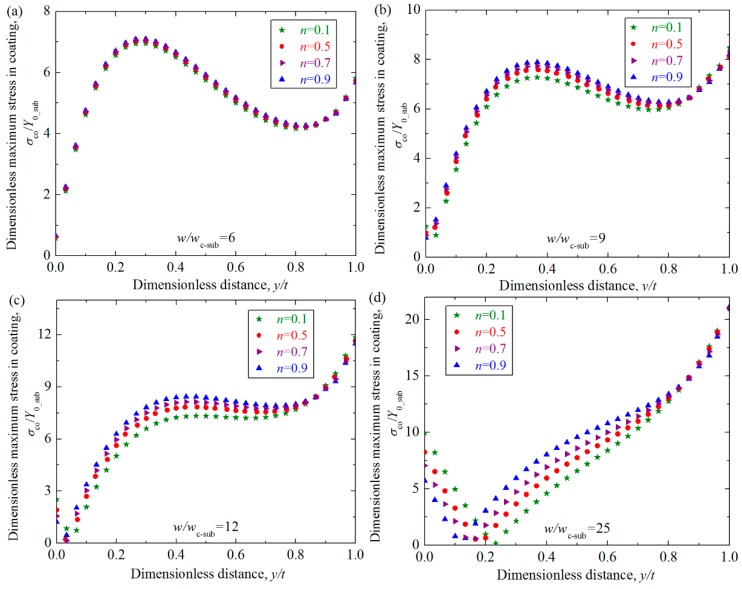
The distribution of the dimensionless equivalent von Mises stresses *σ*_co_*/Y*_0_sub_ in the coating along the axis of symmetry at the different interferences near the inflection point *w*/*w*_c_sub_ = 6, 9, 12, 25 for some typical cases where dimensionless coating thickness *t/R* = 0.01, Young’s modulus ratios *E*_co_*/E*_su_ = 4 at selected hardening exponents of the substrate *n* = 0.1, 0.5, 0.7, 0.9.

An inflection point, where the slope of the increase of maximum stresses changes, appears when the value of *w*/*w*_c_sub_ approximately equals to 9 for all *n* cases as revealed in Figure 8. All curves for different *n* cases consolidate to a single curve when the interferences are less than the inflection point. After the inflection point, the curves separate from each other, and the maximum stress in the coating for larger *n* cases increases more quickly. To study the reason of the appearance of the inflection point, the distribution of the equivalent von Mises stresses *σ*_co_*/Y*_0_sub_ in the coating along the axis of symmetry (*y* axis) was considered at different interferences near the inflection point in Figure 9. It shows that the inflection point appears approximately at dimensionless interference *w*/*w*_c_sub_ = 9 in Figure 8, thus the stress distributions are studied for the cases where interferences range from *w* = 6*w*_c_sub_ to *w* = 25*w*_c_sub_. The geometrical and material parameters were set as those in Figure 8. The dimensionless vertical distance *y*/*t* changes from 0 to 1, meaning the position from the contact surface to the interface between the substrate and the coating. It could be seen from Figure 9 that for the case *w*/*w*_c_sub_ = 6 which is below the inflection point, the maximum stress appears approximately at the dimensionless vertical distance *y*/*t* = 0.28, a position close to the contact surface. For the case *w*/*w*_c_sub_ = 12 and 25 which is above the inflection point, the maximum stress appears at the interface between the substrate and the coating (*y*/*t* = 1). While for the case *w*/*w*_c_sub_ = 9 which is almost the inflection point, the maximum stress appears at two positions: one is at the position *y*/*t* = 0.28 close to the contact area; the other is at the interface. As revealed in Ref. [16], the equivalent von Mises stress in the coating is influenced by two factors: One is the applied contact load and the other is mismatch of elastic modulus. At the position close to the contact surface, the factor of contact load is crucial, while at the interface between the substrate and coating, the factor of the elastic modulus mismatch is dominant. The inflection point appears at the interference where the two factors have the same contributions, leading to two equal maximum stress at different positions.

**Figure 10:**
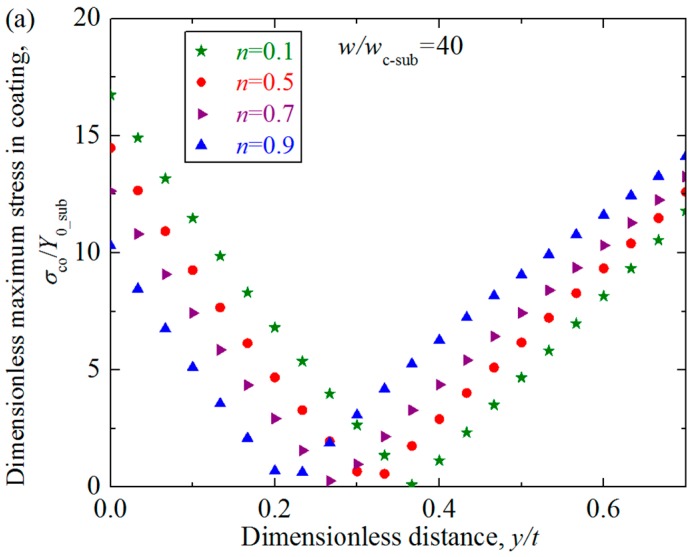

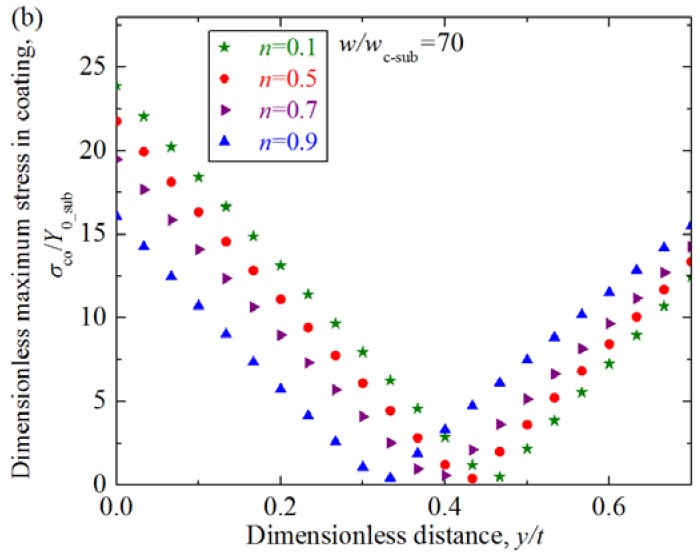
The stress distribution *σ*_co_*/Y*_0_sub_ in the coating along the axis of symmetry in the coating *near the low stress zone* at the dimensionless interferences *w*/*w*_c_sub_ = 40 and 70 for *n* = 0.1, 0.5, 0.7, 0.9 cases when dimensionless coating thickness *t/R* = 0.01, Young’s modulus ratios *E*_co_*/E*_su_ = 4. (**a**) *w*/*w*_c_sub_ = 40; (**b**) *w*/*w*_c_sub_ = 70.

It could be seen from Figure 9 that the distribution and maximum value of the stresses along the axis of symmetry is little affected by the hardening exponent when the interference is small, such as *w*/*w*_c_sub_ less than 9. While as the dimensionless interference *w*/*w*_c_sub_ increases from 12, the distribution of the stress shows to be affected by the values of the substrate hardening exponents to some extent. A notable phenomenon is that as the interference increases, the stress at the position close to the contact surface decreases to zero, which means a low stress zone appears. This phenomenon accords with the conclusion given in Ref. [19]. To show this phenomenon more clearly, Figure 10 presents the stress distribution along the axis of symmetry in the coating for the cases *w*/*w*_c_sub_ = 40 and 70. From Figure 9 and Figure 10, it could also be found that as the interference increases, the low stress zone moves from the position very close to the contact surface (e.g., *w*/*w*_c_sub_ = 12) to the deeper position. Under the same interference, the low stress zone is closer to the contact surface for the larger *n* cases.

## 4. Conclusions

The normal loading process of the contact between a rigid flat and a coated asperity was considered by the FE method in this work. The asperity consisted of the purely elastic coating and the power-law hardening elastic–plastic substrate. The effect of the hardening exponent of the substrate *n* on the contact behaviors was studied in a wide range of interferences. It revealed that the behaviors, including contact load, contact area, coating thickness variation, von Mises stress in the coating, were significantly influenced by the hardening exponent of the substrate. Some conclusions are listed as follows:

(1) The contact load decreases with the decrease of the hardening exponent of the substrate *n* from 1 to 0 at a given interference, meanwhile the contact radius and area enlarges at a special contact load. It is because the substrate (i.e., the asperity) becomes more plastic as the *n* value becomes smaller.
(2) As the interference increases, the coating thickness becomes smaller monotonically for larger hardening exponents such as *n* = 0.9 or larger. While for the smaller *n* cases, the coating thickness recovers gradually after the minimum value. The mechanism should be studied in detail in the future.
(3) The maximum von Mises stress increases as the interference increases. A inflection point appears at the interference where two equal maximum stress values occur at different positions. As the *n* value gets larger, the maximum stress enlarges especially at the moderate and large interference. In addition, the low stress zone appears in the coating during the loading process, and the zone becomes larger as the interference increases. For larger *n* value cases, the low stress zone appears closer to the contact area.

## Nomenclature

*A*	contact area
*A*_c_sub_	critical contact area of the substrate
*C_ν_*	parameter related to *ν*, $C_{\nu }=1.234+1.256\nu$
*E*	Young’s Modulus
*E*_co_	Young’s Modulus of the coating
*E*_su_	Young’s Modulus of the substrate
*n*	strain hardening exponent of the substrate
*P*	normal contact load
*P*_c_sub_	critical load for yield inception of the substrate
*R*	radius of the sphere
*t*	Coating thickness
*w*	interference under normal load
*w*_c_sub_	critical interference for yield inception of the substrate
*Y*_0_sub_	yield strength of the substrate
*ν*	Poisson’s ratio
*ξ*	strain
*σ*	stress
*σ*_max_co_	maximum von Mises stress in the coating
